# Do all Emergency Room Patients With Influenza-like Symptoms Need Blood Cultures? A Retrospective Cohort Study of 2 Annual Influenza Seasons

**DOI:** 10.1093/ofid/ofae242

**Published:** 2024-04-30

**Authors:** Simone Ehrhard, Lukas Herren, Meret E Ricklin, Franziska Suter-Riniker, Aristomenis K Exadaktylos, Wolf Hautz, Martin Müller, Philipp Jent

**Affiliations:** Department of Emergency Medicine, Inselspital, Bern University Hospital, University of Bern, Bern, Switzerland; Department of Emergency Medicine, Inselspital, Bern University Hospital, University of Bern, Bern, Switzerland; Department of Emergency Medicine, Inselspital, Bern University Hospital, University of Bern, Bern, Switzerland; Institute for Infectious Diseases, University of Bern, Bern, Switzerland; Department of Emergency Medicine, Inselspital, Bern University Hospital, University of Bern, Bern, Switzerland; Department of Emergency Medicine, Inselspital, Bern University Hospital, University of Bern, Bern, Switzerland; Department of Emergency Medicine, Inselspital, Bern University Hospital, University of Bern, Bern, Switzerland; Department of Infectious Diseases, Inselspital, Bern University Hospital, University of Bern, Bern, Switzerland

**Keywords:** bacteremia, COVID-19, diagnostic stewardship, flu, influenza

## Abstract

In this retrospective cohort study, we evaluated risk factors for bacteremia in emergency department patients presenting with influenza-like symptoms during influenza epidemic seasons. In patients without fever, chronic heart or chronic liver disease, blood culture collection might be omitted.

Diagnosis of influenza and other respiratory virus infections is based on a syndrome complex including upper respiratory symptoms and often fever, confirmed by a diagnostic test. Common syndromal case definitions, however, have poor sensitivity and specificity to diagnose influenza [1–3]. The sensitivity of the 2011 case definition recommended by the World Health Organization for influenza-like-illness has been reported to be as low as 55%–69% [4, 5]. In addition, signs and symptoms of influenza vary by age, immune status, and presence of underlying comorbidities [6]. Test turnaround times of reverse transcription-polymerase chain reaction in emergency medicine are still too long to exclude alternative differential diagnoses as cause of fever in particular, whereas rapid antigen tests have only low to moderate sensitivity to diagnose influenza [1].

This diagnostic dilemma contributes to a high proportion of blood culture collection in this patient population and may lead to overuse of antibiotics in patients with viral infections [7–9]. However, bacteremia is described to be rare in patients with acute respiratory virus infections: 4.0% (95% confidence interval [CI], 1.9–6.1) of the patients with influenza A, 3.0% (95% CI, 1.2–4.9) with influenza B, and 1.0% (95% CI, .3–1.8) of patients with SARS-CoV-2 infection are bacteremic [10]. Unnecessary collection of blood cultures may harm patients and raise health care costs [11, 12], and inappropriate use of antibiotics has many disadvantages, especially in the view of a rising rate of antibiotic resistance [13].

Therefore, this study aimed to identify risk factors for bacteremia in a cohort of patients presenting with influenza-like symptoms to a tertiary emergency department (ED) during the annual influenza seasons (before the COVID-19 pandemic). In a diagnostic stewardship effort, we tried to identify subpopulations where blood culture sampling can be safely omitted to reduce blood culture collection in patients with suspected viral infection in our ED.

## METHODS

### Study Design

This retrospective cohort study was conducted at the ED of a tertiary Swiss hospital (Inselspital Bern University Hospital, Switzerland) during2 pre-COVID-19 annual influenza seasons according to the Swiss Federal Office of Public Health annual influenza season definition (week 40/2017 to week 16/2018 [October 2, 2017–April 22, 2018]; and week 40/2018 to week 16/2019 [October 1, 2018–April 21, 2019]). All patients aged ≥ 16 years who presented to the ED with influenza-like symptoms and therefore underwent nasopharyngeal swabbing for influenza A and B polymerase chain reaction according to hospital infection prevention and control policy with concurrent blood culture sampling were included.

### Data Collection

Medical data (eg, demographics, clinical complaints, vital values, comorbidities, antibiotic treatment) were extracted from the Eds’ electronic medical record database (Ecare, Turnhout, Belgium). Comorbidities in this retrospective analysis were defined according to US Centers for Disease Control and Prevention health and age factors that are known to increase a person's risk of serious complications from the flu [14]. Blood culture positivity was defined as any blood culture growth, with the exception of known skin contaminants (eg, coagulase negative staphylococci) that were considered contaminants.

### Statistical Analysis

The statistical analysis was performed with Stata 16.1 (StataCorp LLC, College Station, Texas, USA). Depending on normality testing (Shapiro Wilk) median (interquartile range [IQR]) or mean (standard deviation) are shown for continuous variables. The Wilcoxon rank-sum test or unpaired *t* test (for normal distributed variables) was used to compare a continuous variable between positive and negative blood cultures.

Categorical variables were compared between positive and negative blood cultures using chi-squared tests. A *P* value < .05 was considered significant.

A univariable logistic regression analysis was performed with variables with <5% missing values to identify factors associated with blood culture growth. The variables associated with blood culture positivity (*P* < .05) in the univariable logistic regression were further analyzed with a multivariable logistic regression analysis with forward-stepwise selection of the identified variables at a significance level of *P* < .05. The performance of the final model was evaluated using the AUROC metric, where a threshold of 0.7 was considered acceptable for accuracy assessment.

### Ethical Considerations

The study was approved by the regional ethics committee of the Canton of Bern, Switzerland (KEK: 2019-01149). Patients who refused to give general consent for the use of their anonymized data or subsequently withdrew it were excluded from the study.

## RESULTS

### Demographics

During the study period, 1448 patients were tested for influenza; blood cultures were obtained in 546 (37.7%) of these patients. Blood culture positivity in this population was 8.1% (44/546 patients). The median age was 68.0 (IQR 53–77) years and 345 (63.2%) patients were male. The subgroup with a bacteremia had a slightly younger median age with 66.5 (IQR 55–77) years.

Heart disease (n = 150, 27.5%), renal failure (n = 122, 22.3%), and diabetes (n = 111, 20.3%) were the most frequent comorbidities for all patients; for the subgroup with bacteremia, heart disease was significantly more frequent (n = 18, 40.9%, *P* = .037). Diabetes mellitus (n = 12, 27.3%) and renal failure (n = 10, 22.7%) were other common comorbidities in this subgroup. Immunosuppression was present in 177 (32.4%) patients and in 17 (38.6%) patients with bacteremia.

Commonly reported were acute onset of symptoms (symptom duration < 7 days at presentation, n = 440, 80.6%), cough (n = 327, 59.9%), feeling feverish (n = 315, 57.7%), and fatigue (n = 290, 53.1%); patients with bacteremia reported more often feeling feverish (n = 29, 65.9%) and experiencing fatigue (n = 27, 61.4%).

Patients with bacteremia had significantly lower systolic and diastolic blood pressure values and higher temperatures compared with patients without bacteremia (*P* < .001, respectively). The respiratory rate was significantly higher in patients with bacteremia (26 vs 29, *P* = .038), and C-reactive protein and procalcitonin levels were also significantly higher (53 mg/L vs 105 mg/L, *P* = .002 and .27 vs 1.24, *P* = .031, respectively).

Patients with bacteremia were significantly more likely to be hospitalized in the intensive care unit (*P* = .001); 28-day mortality was comparable in both groups (*P* = .969). (Table 1)

**Table 1.** Baseline Characteristics

	Full Cohort			All Patients With Blood Cultures Obtained (Study Population)			No Bacteremia		Bacteremia^a^		
	Total n	(n = 1448)	(%)	Total n	(n = 546)	(%)	(n = 502)	(%)	(n = 44)	(%)	*P*-value
DEMOGRAPHICS											
Sex	1448			546							
Male		881	(60.8)		345	(63.2)	311	(62.0)	34	(77.3)	
Female	…	567	(39.2)	…	201	(36.8)	191	(38.0)	10	(22.7)	.043
Age, median (IQR)	1448	68	(53; 77)	546	68	(53; 77)	68	(53; 77)	66.5	(55; 77)	.864
Age categories, y	1448			546							
16–45	…	239	(16.5)	…	94	(17.2)	89	(17.7)	5	(11.4)	
46–65	…	427	(29.5)	…	160	(29.3)	145	(28.9)	15	(34.1)	
>65	…	782	(54.0)	…	292	(53.5)	268	(53.4)	24	(54.5)	.513
COMORBIDITIES^b^											
Heart disease	1448	407	(28.1)	546	150	(27.5)	132	(26.3)	18	(40.9)	.037
Renal failure	1448	314	(21.7)	546	122	(22.3)	112	(22.3)	10	(22.7)	.949
Diabetes	1448	289	(20.0)	546	111	(20.3)	99	(19.7)	12	(27.3)	.233
Hematological disease	1448	153	(10.6)	546	79	(14.5)	71	(14.1)	8	(18.2)	.465
COPD	1448	216	(14.9)	546	73	(13.4)	65	(12.9)	8	(18.2)	.328
Liver disease	1448	87	(6.0)	546	40	(7.3)	33	(6.6)	7	(15.9)	.023
Asthma	1448	76	(5.2)	546	27	(4.9)	26	(5.2)	1	(2.3)	.394
Obesity (BMI ≥ 40 kg/m^2^)	1448	18	(1.2)	546	7	(1.3)	7	(1.4)	0	(0.0)	0.430
Immunosuppression	1448	393	(27.1)	546	177	(32.4)	160	(31.9)	17	(38.6)	.358
Pregnancy	1448	3	(0.2)	-	…	…	…	…	…	…	
SYMPTOMS											
Symptom duration < 7 d	1448	1048	(74.9)	546	440	(80.6)	400	(79.7)	40	(90.9)	.071
Cough	1448	906	(62.6)	546	327	(59.9)	308	(61.4)	19	(43.2)	.018
Fever feeling	1448	675	(46.6)	546	315	(57.7)	286	(57.0)	29	(65.9)	0.250
Fatigue	1448	730	(50.4)	546	290	(53.1)	263	(52.4)	27	(61.4)	.253
Dyspnea	1448	516	(35.6)	546	179	(32.8)	169	(33.7)	10	(22.7)	.138
Sputum	1448	458	(31.6)	546	165	(30.2)	153	(30.5)	12	(27.3)	.657
Chills	1448	675	(46.6)	546	89	(16.3)	80	(15.9)	9	(20.5)	.437
Headache	1448	226	(15.6)	546	89	(16.3)	84	(16.7)	5	(11.4)	.355
Myalgia	1448	198	(13.7)	546	84	(15.4)	77	(15.3)	7	(15.9)	.920
Sore throat	1448	182	(12.6)	546	72	(13.2)	68	(13.5)	4	(9.1)	.402
Syncope	1448	151	(10.4)	546	60	(11.0)	56	(11.2)	4	(9.1)	.675
Congested nose	1448	189	(13.1)	546	60	(11.0)	57	(11.4)	3	(6.8)	.356
VITAL VALUES AND CLINICAL FINDINGS											
Systolic blood pressure, lowest measurement (mm Hg), mean (SD)	1431	107	(93; 124)	537	105	(22)	106	(22)	93	(20)	<.001
Diastolic blood pressure, lowest measurement (mm Hg), med (IQR)	1430	54	(43; 65)	537	52	(41–64)	54	(42–64)	44	(34–52)	<.001
Lowest GCS, median (IQR)	1443	15	(15; 15)	542	15	(15–15)	15	(15–15)	15	(14–15)	.179
Temperature > 38.0 °C	1448	636	(43.9)	546	317	(58.1)	279	(55.6)	38	(86.4)	<.001
Temperature < 35.0 °C	1448	9	(0.6)	546	3	(0.5)	3	(0.6)	0	-	.607
Highest respiratory rate/min, median (IQR)	1211	26	(22; 30)	466	27	(23–31)	26	(23–31)	29	(25–32)	.038
Lowest oxygen saturation, median (IQR)	1435	92	(89; 95)	540	92	(89–94)	92	(89–94)	91	(88–94)	.319
Oxygen support	1448	554	(38.3)	546	227	(41.6)	209	(41.6)	18	(40.9)	.926
Breath sound abnormal on lung auscultation^c^	1448	641	(44.3)	546	243	(44.5)	222	(44.2)	21	(47.7)	.654
LABORATORY RESULTS											
Leukocyte (g/L), med (IQR)	1412	9.1	(6.1; 12.6)	539	9.5	(5.9–13.9)	9.3	(5.9–13.9)	10.5	(5.2–12.6)	.974
CRP (mg/L), median (IQR)	1416	44	(14; 104)	540	55	(22–120)	53	(20–113.5)	105	(48–194)	.002
Procalcitonin (µg/L), median (IQR)	210	.28	(0.10; 0.87)	104	0.29	(0.10–1.38)	0.27	(0.09–1.12)	1.24	(0.93–2.25)	.031
Creatinine (µmol/L), med (IQR)	1431	81	(64; 108)	542	81	(65–107)	81	(65–106)	80.5	(69–138.5)	.302
Influenza PCR test positive	1448	147	(10.2)	546	58	(10.6)	56	(11.2)	2	(4.5)	.172
X-RAY RESULTS											
Infiltrates detectible in X-ray or CT scan thorax	1448	440	(30.4)	546	194	(35.5)	183	(36.5)	11	(25.0)	.128
OUTCOME											
Hospitalization	1448	1146	(79.1)	546	458	(83.9)	417	(83.1)	41	(93.2)	.080
ICU admission	1448	155	(10.7)	546	75	(13.7)	62	(12.4)	13	(29.5)	.001
28-d mortality	1448	94	(6.5)	546	38	(7.0)	35	(7.0)	3	(6.8)	.969

Abbreviations: BMI, body mass index; COPD, chronic obstructive pulmonary disease; CRP, C-reactive protein; CT, computed tomography; ICU, intensive care unit; IQR, interquartile range; PCR, polymerase chain reaction; SD, standard deviation.

Depending on normality testing (Shapiro Wilk) median (IQR), respectively, mean (SD) are shown for continuous variables, *P* values obtained by Wilcoxon rank-sum test respectively unpaired *t* test. Categorical variables are shown with number (%) in each category, *P* values obtained by chi-squared test.

^a^Corrected for contaminations.

^b^Comorbidities according to US Centers for Disease Control and Prevention health and age factors that are known to increase a person's risk of serious complications from the flu [14]. Liver disease: cirrhosis, chronic hepatitis B, chronic hepatitis C, and liver transplant. Heart disease: congenital heart disease, congestive heart failure, and coronary artery disease. Renal failure: chronic renal failure and kidney transplant Immunosuppression: Compromised immune response due to HIV, hematoncologic malignancy or cancer under chemotherapy or radiation treatment, or immunosuppressive medication.

^c^Abnormal breath sound on lung auscultation includes rales and obstructive breath sounds.

### Risk Factors for Bacteremia in Influenza-like Symptom Patients

Risk factors for bacteremia in the influenza-like symptoms cohort was explored first with univariable logistic regression. Overall, the following factors were associated with bacteremia: chronic liver disease (OR 2.69, CI 1.11; 6.49, *P* = .028) and chronic heart disease (OR 1.94, CI 1.03; 3.65, *P* = .040) as comorbidities. Furthermore, findings like body temperature > 38.0°C (OR 5.06, CI 2.10; 12.19, *P* < .001), highest respiratory rate (odds ratio [OR], 1.06; CI, 1.00˗1.11; *P* = .031), and lowest systolic blood pressure (OR, 0.97; CI, .96˗.99; *P* < .001) during the ED stay were associated with a higher probability of bacteremia, whereas reported cough (OR, .48; CI, .26˗.89; *P* = .020) was associated with a lower probability of bacteremia.

Second, a stepwise multivariable logistic regression was performed, including variables associated in the univariable analysis with positive blood cultures. Temperature > 38.0 °C (OR, 6.39; CI, 2.33–17.49), liver disease (OR, 5.62; CI, 1.91–16.53), and heart disease (OR, 4.68; CI, 2.11–10.38) were the factors found with the highest association with bacteremia (further factors are presented in Table 2).

**Table 2.** Multivariable Logistic Regression, Risk Factors for Bacteremia in Patients With Flu-like Symptoms; Variables Associated With Positive Blood Culture With *P* < .05

Bacteremia	Odds Ratio	95% Confidence Interval	*P* > z
Temperature > 38. 0°C	6.39	2.33–17.49	<.001
Liver disease	5.62	1.91–16.53	.002
Heart disease	4.68	2.11–10.38	<.001
CRP per mg/L^a^	1.01	1.00–1.01	<.001
Systolic blood pressure, lowest measurement, per mm Hg^b^	0.97	0.96–0.99	.007
Cough	0.39	0.19–0.81	.011

Abbreviation: CRP, C-reactive protein.

Number of observations n = 523. Area under receiver operating characteristic curve: 0.843.

^a^The odds ratio of CRP was calculated for each increase in 1 mg/L.

^b^The odds ratio of systolic blood pressure, lowest measurement, was calculated per decrement of 1 mm Hg.

In the subgroup of blood cultures taken from patients with absence of any of these 3 predictors of bacteremia (n = 140), only 1.4% (n = 2) of the blood cultures were positive, whereas in patients with at least 1 of the mentioned predictors (n = 406), blood culture positivity was 10.3% (n = 42) (OR, 7.96; 95% CI, 2.02–68.62; *P* < .001). In patients without the 2 identified comorbidities heart and liver disease (n = 360), blood culture positivity was 5.3% (19/360 patients), whereas in patients with heart or liver disease or both, blood culture positivity was 13.4% (25/186) (OR, 2.79; 95% CI, 1.43–5.51; *P* < .001). In patients with all of the 3 identified predictors (n = 24), blood culture positivity was 29.2% (7/24 patients) (OR, 5.40; 95% CI, 1.77–14.72; *P* < .001).

## DISCUSSION

During 2 influenza epidemic seasons before the COVID-19 pandemic, bacteremia was detected in only 8.1% of patients presenting with influenza-like symptoms in a tertiary ED, which is comparable to an earlier report [15]. Our retrospective data suggest that the collection of blood cultures could be omitted in patients presenting with influenza-like symptoms during influenza season that have neither fever nor chronic heart or liver disease. However, this finding should be prospectively validated. A meaningful score to identify patients without bacteremia in febrile or hypothermic patients presenting with influenza-like symptoms could not be derived from the retrospective dataset of this study. In addition, the identified predictors of bacteremia might be less reliable in elderly patients with a suspicion of influenza, where fever is not as commonly present even in bacteremia. The predictors found in our study have some overlap with predictors for bacteremia identified in a general ED population (not focusing on influenza-like symptom subpopulation), but in that broader population the association with the comorbidities heart disease or liver diseases was not mentioned [16].

Febrile illness with suspected blood stream infection is a leading cause for hospital admission [17, 18], and obtaining blood cultures is a common practice during initial ED presentation of patients who may have an infection [12]. It has been shown that physicians overestimate the likelihood of bacteremia in patients in general [19, 20]. In high-income countries, blood stream infection has been documented in only 1.4% to 8.3% of blood cultures taken from patients presenting to EDs [12, 21–23]. In contrast, rapid identification of patients at risk for bacteremia is critical in the ED because untreated bacteremia can lead to sepsis and septic shock with an estimated mortality rate of 30% to 50% [12, 17, 24–27]. Therefore, the ED is an important setting for diagnostic and therapeutic stewardship approaches [11].

The proportion of positive blood cultures both in patients with an influenza infection, and, more recently, with a SARS-CoV-2 infection, is low, and in the mentioned subgroups it might be low enough to justify omitting blood culturing.

Our study has several limitations. It was performed in a retrospective cohort from before the COVID-19 pandemic; the findings of this study should be confirmed in a mixed SARS-CoV-2 and influenza respiratory virus infection season in the future. Nevertheless, observed blood culture positivity in COVID-19 patients are in a comparable range to influenza patients. Therefore, the results might be transferable to a more current setting. Furthermore, blood cultures were obtained in only about 40% of all patients with influenza-like symptoms, and the results of the subpopulation studied may differ from those of the total population because of potential selection bias.

## CONCLUSION

Bacteremia is rarely found in ED patients presenting with influenza-like symptoms during epidemic seasons, especially in the absence of identified predictors such as fever and chronic heart and liver disease. In the sense of a diagnostic stewardship approach, blood culture collection could be omitted in a relevant proportion of patients presenting with flu-like symptoms during the annual epidemic season. Identified risk factors for bacteremia should be externally validated, ideally in a prospective cohort.

## Notes

***Author Contributions***. All authors have contributed substantially to conception and design of the study. L. H. did the manual coding of the data. M. M. performed the analysis. S. E., L. H., and P. J. did the interpretation of the data supported by M. M. S. E., L. H., and P. J. drafted the manuscript and M. R., F. S., A. E., W. H., and M. M. revised it critically. All approved the final version to be published.

This research was financed by the contributing departments, without additional external funding sources. The authors declare that they have no conflict of interest.

***Data availability***. The datasets used and/or analyzed during the current study are available from the corresponding author on reasonable request.

***Ethics approval and***  ***Patient Consent Statement***. The study was approved by the regional ethics committee of the Canton of Bern, Switzerland. Patients who refused to give the general consent for the use of their anonymized data were excluded from the study (KEK: 2019-01149).

Consent for publication. Not applicable.

***Financial support*.** No external funding.

## References

[1] Dugas AF, Valsamakis A, Atreya MR, et al Clinical diagnosis of influenza in the ED. Am J Emerg Med. 2015; 33:770–5. 25827595 10.1016/j.ajem.2015.03.008 PMC4458223

[2] Falsey AR, Baran A, Walsh EE. Should clinical case definitions of influenza in hospitalized older adults include fever? Influenza Other Respir Viruses 2015; 9(Suppl 1):23–9. 26256292 10.1111/irv.12316 PMC4549099

[3] Casalegno JS, Eibach D, Valette M, et al Performance of influenza case definitions for influenza community surveillance: based on the French influenza surveillance network GROG, 2009–2014. Euro Surveill 2017; 22:30504. 28422004 10.2807/1560-7917.ES.2017.22.14.30504 PMC5388124

[4] Ong AK, Chen MI, Lin L, et al Improving the clinical diagnosis of influenza–a comparative analysis of new influenza A (H1N1) cases. PLoS One 2009; 4:e8453. 20041115 10.1371/journal.pone.0008453 PMC2795196

[5] Centers for Disease Control and Prevention. Overview of influenza surveillance in the United States. 2013. Available at: http://www.cdc.gov/flu/pdf/weekly/overview.pdf. Accessed 21 November 2013.

[6] Centers for Disease Control and Prevention . Available at: https://www.cdc.gov/flu/professionals/diagnosis/labrolesprocedures.htm

[7] Klein EY, Monteforte B, Gupta A, et al The frequency of influenza and bacterial coinfection: a systematic review and meta-analysis. Influenza Other Respir Viruses 2016; 10:394–403. 27232677 10.1111/irv.12398 PMC4947938

[8] Gonzales R, Bartlett JG, Besser RE, et al Principles of appropriate antibiotic use for treatment of acute respiratory tract infections in adults: background, specific aims, and methods. Ann Intern Med 2001; 134:479–86. 11255524 10.7326/0003-4819-134-6-200103200-00013

[9] Metlay JP, Camargo CA, MacKenzie J, et al Cluster-randomized trial to improve antibiotic use for adults with acute respiratory infections treated in emergency departments. Ann Emerg Med 2007; 50:221–30. 17509729 10.1016/j.annemergmed.2007.03.022

[10] Thelen JM, Buenen AGN, van Apeldoorn M, Wertheim HF, Hermans MHA, Wever PC. Community-acquired bacteraemia in COVID-19 in comparison to influenza A and influenza B: a retrospective cohort study. BMC Infect Dis 2021; 21:199. 33618663 10.1186/s12879-021-05902-5 PMC7897875

[11] Boyles TH, Davis K, Crede T, Malan J, Mendelson M, Lesosky M. Blood cultures taken from patients attending emergency departments in South Africa are an important antibiotic stewardship tool, which directly influences patient management. BMC Infect Dis 2015; 15:410. 26437651 10.1186/s12879-015-1127-1 PMC4594638

[12] Shapiro NI, Wolfe RE, Wright SB, Moore R, Bates DW. Who needs a blood culture? A prospectively derived and validated prediction rule. J Emerg Med 2008; 35:255–64. 18486413 10.1016/j.jemermed.2008.04.001

[13] Aslam B, Wang W, Arshad MI, et al Antibiotic resistance: a rundown of a global crisis. Infect Drug Resist 2018; 11:1645–58. 30349322 10.2147/IDR.S173867 PMC6188119

[14] Anderson KB, Simasathien S, Watanaveeradej V, et al Clinical and laboratory predictors of influenza infection among individuals with influenza-like illness presenting to an urban Thai hospital over a five-year period. PLoS One 2018; 13:e0193050. 29513698 10.1371/journal.pone.0193050 PMC5841736

[15] Kongre VA, Pol SS, Bharadwaj RS, et al Bacteriological study among influenza-like illness cases in a community setting in Pune, India. Cureus 2018; 10:e3601. 30680262 10.7759/cureus.3601 PMC6338396

[16] Su CP, Chen TH, Chen SY, et al Predictive model for bacteremia in adult patients with blood cultures performed at the emergency department: a preliminary report. J Microbiol Immunol Infect 2011; 44:449–55. 21684227 10.1016/j.jmii.2011.04.006

[17] Angus DC, Linde-Zwirble WT, Lidicker J, Clermont G, Carcillo J, Pinsky MR. Epidemiology of severe sepsis in the United States: analysis of incidence, outcome, and associated costs of care. Crit Care Med 2001; 29:1303–10. 11445675 10.1097/00003246-200107000-00002

[18] Reddy EA, Shaw AV, Crump JA. Community-acquired bloodstream infections in Africa: a systematic review and meta-analysis. Lancet Infect Dis 2010; 10:417–32. 20510282 10.1016/S1473-3099(10)70072-4 PMC3168734

[19] Makadon HJ, Bor D, Friedland G, Dasse P, Komaroff AL, Aronson MD. Febrile inpatients: house officers’ use of blood cultures. J Gen Intern Med 1987; 2:293–7. 3655954 10.1007/BF02596161

[20] Poses MARM . When you hear hoofbeats: physicians overvalue recent experience when diagnosing bacteremia [abstract]. Clin Res. 1989; 37 :781A.

[21] Howie N, Gerstenmaier JF, Munro PT. Do peripheral blood cultures taken in the emergency department influence clinical management? Emerg Med J 2007; 24:213–4. 17351231 10.1136/emj.2006.039875 PMC2660034

[22] Kelly AM . Clinical impact of blood cultures taken in the emergency department. J Accid Emerg Med 1998; 15:254–6. 9681310 10.1136/emj.15.4.254 PMC1343139

[23] Mountain D, Bailey PM, O'Brien D, Jelinek GA. Blood cultures ordered in the adult emergency department are rarely useful. Eur J Emerg Med 2006; 13:76–9. 16525233 10.1097/01.mej.0000188231.45109.ec

[24] Brun-Buisson C . The epidemiology of the systemic inflammatory response. Intensive Care Med 2000; 26(Suppl 1):S64–74. 10786961 10.1007/s001340051121 PMC7094973

[25] Sands KE, Bates DW, Lanken PN, et al Epidemiology of sepsis syndrome in 8 academic medical centers. Jama 1997; 278:234–40. 9218672

[26] Rivers E, Nguyen B, Havstad S, et al Early goal-directed therapy in the treatment of severe sepsis and septic shock. N Engl J Med 2001; 345:1368–77. 11794169 10.1056/NEJMoa010307

[27] Rangel-Frausto MS, Pittet D, Costigan M, Hwang T, Davis CS, Wenzel RP. The natural history of the systemic inflammatory response syndrome (SIRS). A prospective study. JAMA. 1995; 273:117–23. 7799491

